# Erratum for Snyder et al., “Performance of the LifeScale automated rapid phenotypic antimicrobial susceptibility testing on Gram-negative rods directly from positive blood cultures”

**DOI:** 10.1128/jcm.00156-25

**Published:** 2025-02-12

**Authors:** James W. Snyder, Nadia Chaudhry, Wesley Hoffmann

## ERRATUM

Volume 62, no. 12, e00922-24, 2024, https://doi.org/10.1128/jcm.00922-24. Page 2, lines 11–13: The sentence “Additional phenotypic AST systems awaiting FDA clearance for the direct testing of positive blood cultures include the Vitek REVEAL (bioMerieux)” should be deleted. As mentioned earlier in the paragraph, this platform does have FDA clearance.

